# Environmental and occupational respiratory diseases – 1048. Association of VDR SNPS with allergy asthma in Colombian Caribbean patients. A pilot study

**DOI:** 10.1186/1939-4551-6-S1-P47

**Published:** 2013-04-23

**Authors:** Gloria Garavito, Mario Sanchez Borges, Elkin Navarro, Eduardo Egea, Elkin Navarro

**Affiliations:** 1 División Ciencias De La Salud, Universidad Del Norte, Barranquilla, Colombia; 2 Allergy and Clinical Immunology, Clinica El Avila, Caracas, Venezuela

## Background

VDR gene maps to chromosome 12q, region q13-23 near a region commonly linked to asthma. The VDR hence is good candidate to be investigated for association with asthma. In this study, we analyzed the association between SPNs VDR and asthma susceptibility in a sample of asthmatic patients living in a Colombian Caribbean area.

## Methods

Blood samples were collected from 37 asthmatics patients and 60 unrelated normal individuals with informed consent. The DNA was isolated from PBL by using a salting out technique. SNPs were selected from dbSNP: http://www.ncbi.nlm.nih.gov/SNP/. Nine VDR polymorphisms were genotyped : FokI (rs2228570), Apal (rs7975232), BsmI (rs1544410) , TaqI (rs731236), rs703842, rs4516035, rs 2248098, rs 71139166, rs7975232.

Total IgE was determined with a commercial ELISA kit .The allergens tested on patients by Prick Test were D Pteronyssinus, D Farine B Tropicalis and P Americana, Genotyping was performed using MALDI-TOF mass spectrometry of allele-specific primer extension products generated from amplified DNA sequences (Mas-sARRAY, SEQUENOM Inc).

## Results

There was no statistically significance difference in allelic frequencies of TaqI (rs731236), and rs2248098.The patients rs7975232 BSMI ( OR 4,45 p=0.002) and rs1544410 ( OR 3,16 p= 0.013 ) polymorphism demonstrated significant association as a susceptibility markers. The alleles rs4516035 (OR 0.4 p= 0.090) and rs7139166 Apa I ( OR 0.41 p=0.064) was observed to be significantly associated as a protective markers .

The FokI rs2228570 marker was excluded from the analysis (Hardy-Weinberg disequilibrium failed).

There was no difference in the serum levels of 25 hydroxyvitaminD3 between patients(mean=46,18ng/ml) and controls. (mean=51.95ng/ml); p= 0,15 .Levels of IgE was higher in the patients ( mean=262.9UI/ml) compared with controls( mean=162.03UI/ml); p= 0.002. It wasn´t significant difference between levels of vitamin D and total IgE (p=0,852).

## Conclusions

Although a association of the VDR variants to patients with asthma could not be asserted, our results raise the possibility of a susceptibility association in rs7975232 BSMI and rs1544410 in this group of Caribbean patient’s population. This is the first work set up in the Colombian population. More data from family studies on this admixed population should be done to validate this possible association.

